# Adjusting the duration of androgen deprivation therapy (ADT) based on nadir PSA for high risk localized prostate cancer patients treated with definitive external beam radiation therapy and ADT

**DOI:** 10.1186/s12894-022-01145-x

**Published:** 2022-12-12

**Authors:** Zeina Ayoub, Jamal Khader, Muhammad Bulbul, Raja B. Khauli, Therese Y. Andraos, Ali Shamseddine, Deborah Mukherji, Fady B. Geara

**Affiliations:** 1 Department of Radiation Oncology, The Naef K. Basile Cancer Institute, The American University of Beirut Medical Center, Bliss Street, Riad El Solh, Beirut, 11072030 Lebanon; 2 Department of Radiation Oncology, King Hussein Cancer Center, Amman, Jordan; 3 Division of Urology, The American University of Beirut Medical Center, Beirut, Lebanon; 4 Division of Medical Oncology, The Naef K. Basile Cancer Institute at the American University of Beirut Medical Center, Beirut, Lebanon; 5 Present Address: Oncology Institute Cleveland Clinic Abu Dhabi, Al Maryah Island, Abu Dhabi, United Arab Emirates

**Keywords:** Prostate cancer, Nadir PSA, Androgen deprivation therapy

## Abstract

**Background:**

A nadir Prostate-Specific Antigen (nPSA) of 0.06 ng/mL has been shown to be a strong independent predictor of biochemical recurrence-free survival (bRFS) in patients with intermediate or high-risk (HR) prostate cancer treated with definitive external beam radiation therapy (RT) and androgen deprivation therapy (ADT). We aimed to examine the association between the duration of ADT and bRFS in HR localized prostate cancer, based on nPSA.

**Methods:**

Between 1998 and 2015, 204 patients with HR localized prostate cancer were identified. Of them, 157 patients (77.0%) reached the desired nPSA of < 0.06 ng/mL (favorable group), while 47 (23.0%) did not (unfavorable group). Duration of ADT varied among patients depending on physician preference, patient tolerance, and/or compliance. Survival outcomes were calculated using Kaplan–Meier methods and predictors of outcomes using multi-variable cox regression model.

**Results:**

In the favorable group, ADT for at least 12 months lead to superior bRFS compared to ≤ 9 months of ADT (*P* = 0.036). However, no significant difference was seen when examining the value of receiving ADT beyond 12, 18, or 24 months, respectively. On univariate analysis for bRFS, the use of ADT for at least 12 months was significant (*P* = 0.012) as well as time to nadir PSA (tnPSA), (≤ 6 vs > 6 months); (*P* = 0.043). The presenting T stage was borderline significant (HR 3.074; 95% CI 0.972–9.719; *P* = 0.056), while PSA at presentation, Gleason Score and age were not. On multivariate analysis, the use of ADT for 12 months (*P* = 0.012) and tnPSA (*P* = 0.037) remained significant. In the unfavorable group, receiving ADT beyond 9 and 12 months was associated with improved bRFS (*P* = 0.044 and 0.019, respectively). However, beyond 18 months, there was no significant difference.

**Conclusion:**

In HR localized prostate cancer patients treated with definitive RT and ADT, the total duration of ADT may be adjusted according to treatment response using nPSA. In patients reaching a nPSA below 0.06 ng/mL, a total of 12 months of ADT may be sufficient, while in those not reaching a nPSA below 0.06 ng/mL, a total duration of 18 months is required.

## Background

Androgen deprivation therapy (ADT) combined with external beam radiation therapy (EBRT) is an effective primary treatment for patients with high-risk (HR) prostate cancer. Several prospective randomized controlled trials evaluating the value of ADT have shown an improved disease-specific survival and overall survival compared to radiation therapy alone [1–3]. ADT is usually given in the neoadjuvant, concurrent and adjuvant setting in HR prostate cancer, and consists of a luteinizing hormone-releasing hormone (LHRH) agonist, an LHRH agonist with a first-generation antiandrogen, or an LHRH antagonist.

Multiple studies have favored long-term over short-term ADT in patients with HR prostate cancer [4–9], and a recent meta-analysis showed that prolonged duration of ADT is associated with improved survival outcomes in patients with Gleason scores of 8–10 [10]. ADT is associated with a variety of adverse effects including sexual dysfunction, hot flashes, osteoporosis and increased risk of fractures, obesity, insulin resistance and a greater risk of diabetes and cardiovascular diseases, among others [11–14]. The risk of adverse events increases with the duration of treatment which affects patients’ well-being and quality of life, and the best way to mitigate the side effects is by shortening the duration of treatment [15].

We therefore aimed to determine whether the duration of ADT can be adjusted according to response to definitive EBRT and ADT in hopes of selecting a subgroup of patients with favorable prognosis who may not require prolonged use of ADT. Several studies have shown that the nadir PSA (nPSA)—i.e. the lowest level of PSA achieved after completion of radiation, is a determinant of outcomes, and we have previously shown that a nPSA of 0.06 ng/mL is a strong independent predictor of biochemical recurrence-free survival (bRFS) in patients with intermediate-risk (IR) or HR prostate cancer treated by definitive EBRT and ADT [16]. Here, we aimed to examine the association between the duration of ADT and bRFS in HR localized prostate cancer patients, and determine the optimal ADT duration according to nPSA, by using the previously reported nPSA cut-off of 0.06 ng/mL.

## Methods

### Study population

This is an Institutional review boards-approved, multi-institutional review of prostate cancer patients treated between January 1998 and July 2015. The study included two patient populations treated at two institutions with existing collaborative research programs: the Naef K Basile Cancer Institute (NKBCI) at The American University of Beirut, Lebanon and the King Hussein Cancer Center (KHCC) Amman, Jordan. Our initial study population consisted of 375 patients with IR and HR prostate cancer [16]. In our current study and analysis, we only selected the group of patients with HR prostate cancer treated with a combination of EBRT and ADT. HR prostate cancer was defined as per the National Cancer Center Network definitions as having one or more of the following high risk factors: clinical stage T3a-T4, Gleason score 8–10, and PSA > 20 ng/mL [17]. A total of 235 patients fit these criteria. We excluded 31 patients with missing nPSA value. Of the remaining 204 HR prostate cancer patients receiving bimodality treatment and with available information on the value and date of nPSA, 157 patients reached a nPSA < 0.06 ng per mL (77%), while 47 patients had a nPSA ≥ 0.06 ng per mL (23%) and constituted the favorable and unfavorable cohorts, respectively.

### Treatment details

ADT included an LHRH agonist in all patients, which was given alone or in combination with anti-androgen therapy. The duration of ADT varied according to physician preference and was based on published reports on this subject in the treatment era, patient tolerance, and patient compliance. Combination ADT was not commonly practiced and followed physician’s discretion. In this retrospective review, only 36 patients (18%) received combined ADT with LHRH agonists and antiandrogens, and the remainder 82% received LHRH agonists alone. All PSA measurements were done at AUBMC and KHCC to decrease variability across different laboratories and have a more reliable comparison of different PSA values for each patient. The mean time to start RT was 105 days (IQR 75–139 days). Radiation therapy CT simulation was done in a supine position, with full bladder. Treatment planning was done using 3D-Conformal Radiation Therapy (3D-CRT) or Intensity-Modulated Radiation Therapy (IMRT) with daily image-guided radiation therapy (IGRT). The choice between 3D-CRT and IMRT depended on patient insurance coverage. Conformal 3D-CRT was used in 109 patients (53.4%) and IMRT was used in 95 patients (46.6%). No treatment with brachytherapy was given. Treatment doses ranged between 70 and 78 Gy, in 2 Gy per fraction. Treatment target included the prostate and seminal vesicles, and organs at risk included the rectum, bladder and femoral heads. No patients received radiation therapy to the pelvic nodal regions. PSA levels after ADT and EBRT were typically obtained every 4 months for the first 2 years and every 6 months thereafter. These values were recorded, and the lowest PSA value attained was considered as the nPSA. Median time to nPSA was defined as time from the end of EBRT until nPSA was achieved. Biochemical recurrence was defined as “nPSA + 2 ng/mL” based on the Phoenix definition [18]. Time to biochemical recurrence was calculated from the end of EBRT until time of recurrence.

### Statistical analysis

Statistical analyses were performed using SPSS 23.0 (SPSS Inc., Chicago, IL, USA). Baseline patient and clinical characteristics of the two cohorts were analyzed and compared using chi-square testing for categorical variables and independent sample t-test for continuous variables. Survival outcomes were calculated using Kaplan–Meier methods and compared using the log-rank test. Predictors of outcomes were analyzed using multi-variable cox regression model, first for the entire cohort of high-risk patients, followed by a subgroup analysis based on nPSA. *P* value < 0.05 was considered statistically significant.

## Results

### Tumor characteristics and treatment details

A total of 204 patients constituted this study’s patient cohort. The median age for this cohort was 70 years (Interquartile range (IQR) 65–74 years). Table 1 shows patient and tumor characteristics stratified according to the PSA nadir value. There were no statistically significant differences in the baseline age, Gleason score, stage or PSA at presentation between the 2 groups. Of note, the unfavorable group had a numerically higher percentage of patients with more advanced T stage, higher PSA at presentation and more GS 8–10, but these differences were not statistically significant (*P* = 0.10, 0.12, 0.25 respectively; Table 1). This discrepancy in number and baseline characteristics between favorable and unfavorable groups could be due to the fact that more of the unfavorable patients had multiple high-risk features combined than the favorable patients. However, both groups had a sufficient number of events, and this relative imbalance in tumor characteristics was addressed in multivariate analyses and found to be of no effect. The mean duration of ADT was 21.5 months in the favorable group, and 20.2 months in the unfavorable group (*P* = 0.493). The median duration for ADT was 24.0 months (IQR: 9–36) for the favorable group and 18.5 months (IQR: 8–36) for the unfavorable group. There was no significant correlation between the total duration of ADT and the presence of comorbidities, such as diabetes mellitus (*P* = 0.547), hypertension (*P* = 0.902) and coronary artery disease (*P* = 0.347). There were no significant differences in PSA levels and nPSA between AUBMC and KHCC treated patients.

**Table 1:** Patient and tumor characteristics (n = 204)

	Nadir PSA < 0.06 ng/mL (n = 157)		Nadir PSA ≥ 0.06 ng/mL (n = 47)		*P* value
	N	%	N	%	
Age: ≤ 70 years	86	54.8	26	55.3	0.948
> 70 years	71	45.2	21	44.7	
GS: ≤ 7	68	43.3	16	34.0	0.257
8–10	89	56.7	31	66.0	
Stage: T1-T2	93	60.4	21	46.7	0.102
T3–T4	61	39.6	24	53.3	
PSA at presentation: ≤ 20 ng/mL	83	53.2	19	40.4	0.125
> 20 ng/mL	73	46.8	28	59.6	
*Duration of ADT (months)*					
Mean	21.5		20.2		0.493
Median (IQR)	24.0 (9–36)		18.5 (8–36)		
Duration of ADT					
≤ 6 months	36 (23.4)		10 (21.7)		0.495
≤ 9 months	39 (25.3)		13 (28.3)		0.412
≤ 12 months	48 (31.2)		15 (32.6)		0.494
≤ 18 months	54 (35.1)		24 (52.2)		0.028
≤ 24 months	103 (66.9)		28 (60.9)		0.280
> 24 months	51 (33.1)		18 (39.1)		0.280
Missing	3 (1.9%)		1 (2.1%)		
Mean RT dose (Gy)	73.8 (Range 70–78)		73.1 (Range 70–78)		0.113
Time to nPSA (months Median (IQR)	6.0 (3.2–10.0)		N/A*		

*Patients in the unfavorable group did not reach nPSA of 0.06 ng/mL

### Biochemical recurrence-free survival outcomes

At a median follow-up of 42.9 months (IQR 24.8–76.6 months), the 2, 5 and 10-year bRFS rates for the total cohort of 204 patients with HR prostate cancer were 97.1%, 86.5% and 65.9%, respectively. A total of 28 patients (13.7%) developed a biochemical recurrence; 13 (8.3%) in the favorable group and 15 (39.1%) of the unfavorable group. The bRFS was significantly higher in patients who achieved an nPSA < 0.06 ng/mL (*P* < 0.001); For the favorable group, at a median follow-up of 42.5 months (IQR 24.9–79.2 months), the 2, 5 and 10-year bRFS rates were 99.2%, 93.5% and 75.9%, respectively. For the unfavorable group, at a median follow-up of 44.8 months (IQR 21.3–65.6 months), the 2, 5 and 10-year bRFS rates were 89.6%, 64.9% and 55.7%, respectively.

### Total duration of ADT and bRFS

**Fig. 1:**
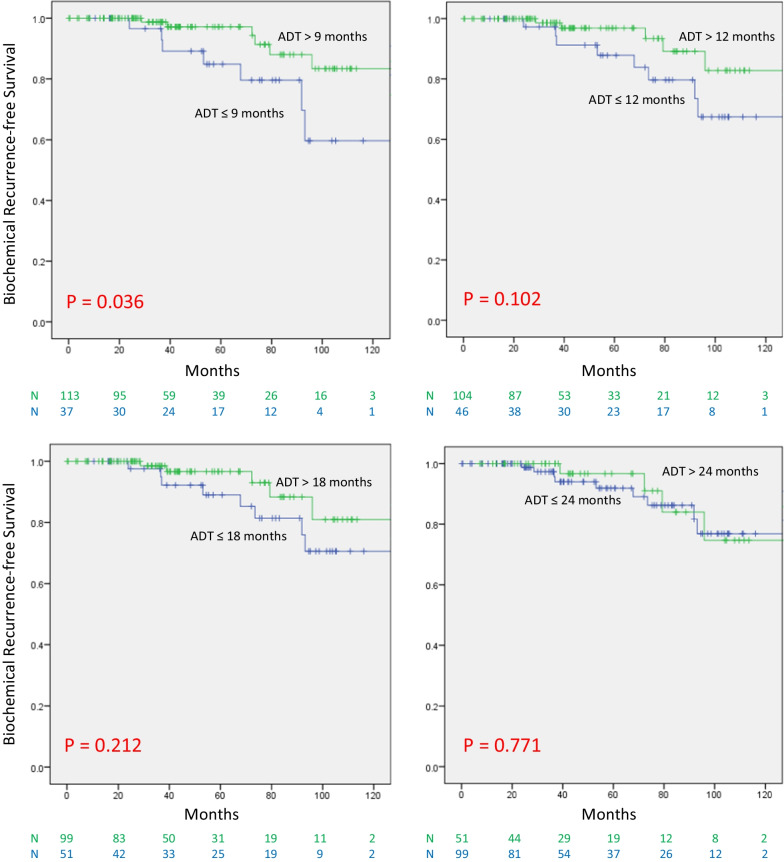
bRFS in the favorable group according to the duration of ADT

**Fig. 2:**
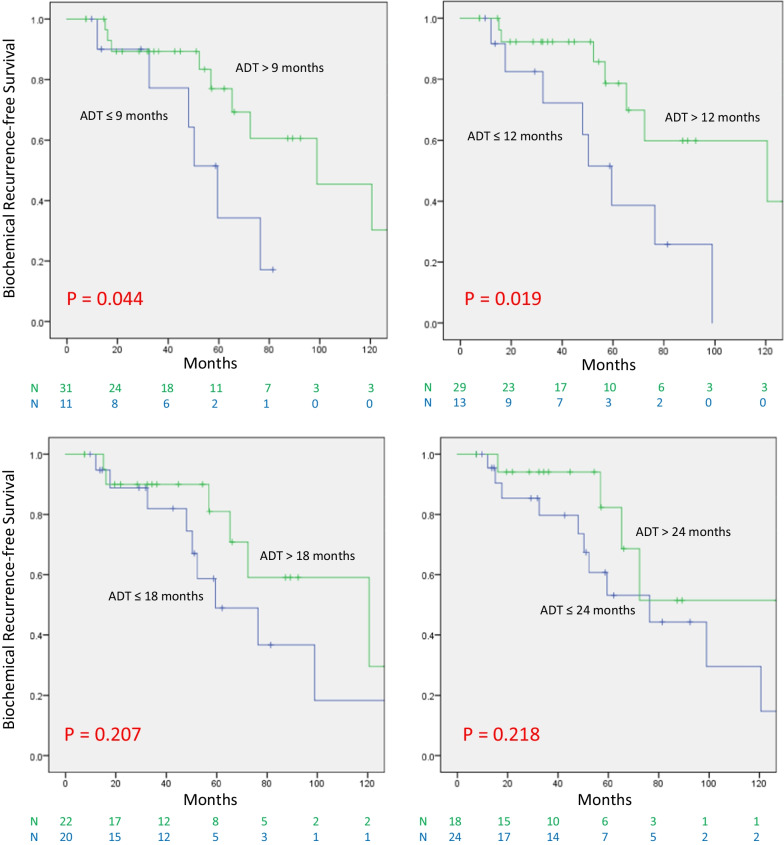
bRFS in the unfavorable group according to the duration of ADT

We examined the association between the total duration of ADT and bRFS. We examined the duration of ADT in 3 months increments starting with 6 and ending at 24 months. The choice of this 3-months increment was based on most clinical ADT protocols which go between 6 months for intermediate-risk to 18–24 months for HR patients. In the favorable group (Fig. 1), the use of ADT for more than 9 months lead to superior bRFS compared to those receiving ≤ 9 months of ADT therapy (*P* = 0.036). However, no significant difference in bRFS was seen when examining the value of receiving ADT beyond 12, 18 and 24 months, respectively, indicating that the use of ADT beyond 12 months did not improve bRFS in this group of patients. The analysis was repeated for patients in the unfavorable group (who failed to reach the nPSA target of 0.06 ng/mL), (Fig. 2). In this group of patients, receiving ADT beyond 9 and 12 months was associated with an improved bRFS compared to those receiving ADT for less than that (*P* = 0.044 and 0.019, respectively). However, this lost significance when ADT was extended for more than 18 months compared to those receiving ADT for 18 months or less. Similarly, no association was found between bRFS and the use of ADT beyond 24 months.

### Time to reach nPSA and bRFS

**Fig. 3:**
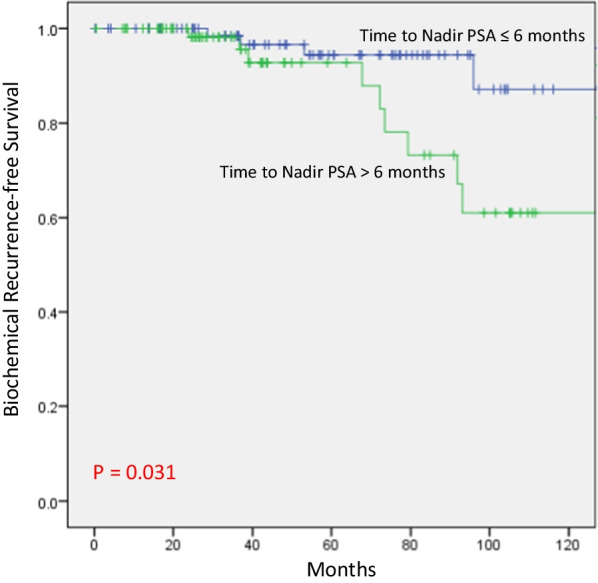
bRFS in the favorable group according to time to Nadir

**Fig. 4:**
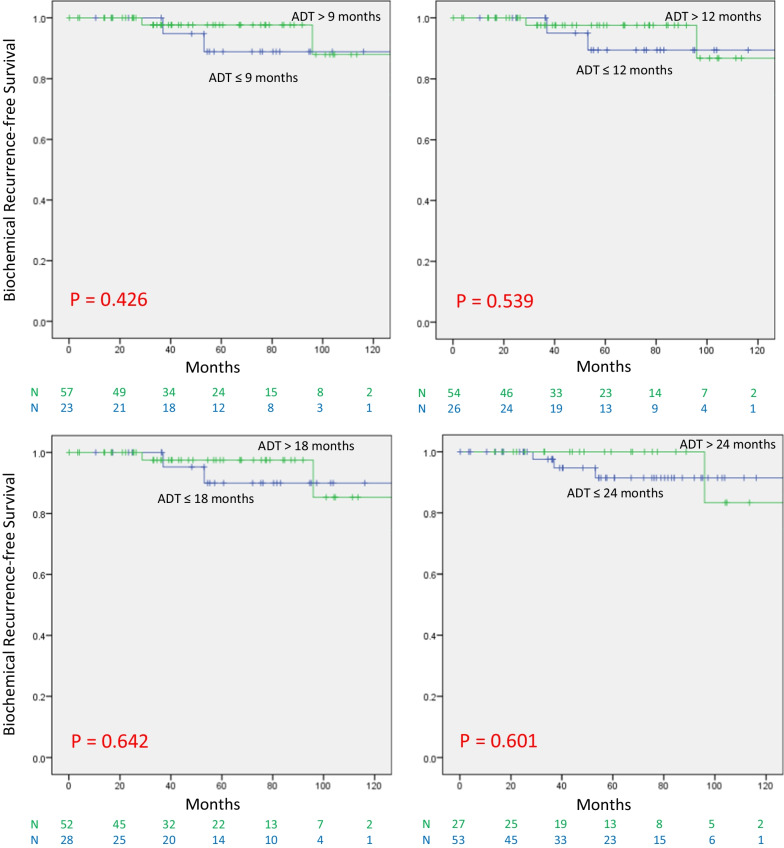
bRFS in the favorable group AND a tnPSA < 6 months, according to ADT duration

For the total cohort, the mean time to nPSA (tnPSA) was 9.8 months (range 1.0–108.0 months), and median tnPSA was 6.6 months (IQR 3.8–11.9 months). For the favorable group, the mean tnPSA was 8.3 months (range 182 1.3–31.0 months) and median tnPSA was 6.0 months (IQR 3.2–10.0 months). For the unfavorable group the mean tnPSA was 14.8 months (range 0.9–108 months) and median tnPSA was 9.4 months (IQR 6.0–17.0 184 months). The means for tnPSA between both groups were significantly different (*P* = 0.023). When dichotomized according to the median tnPSA, the 10-year bRFS in the group with tnPSA ≤ 6 months was 87.1%, compared to 61.0% for those with tnPSA > 6 months (*P* = 0.031), (Fig. 3). To assess the relative effect of tnPSA and nPSA on bRFS, we then examined the effect of the duration of ADT on bRFS in this very favorable group of patients who did achieve the nPSA < 0.06 ng/mL in ≤ 6 months. The duration of ADT did not affect bRFS in this very favorable group of patients (Fig. 4).

### Predictors of outcome

A univariate analysis assessing predictors of biochemical recurrence was initially done for the entire cohort, and included GS, PSA at presentation, age, T-stage, nPSA and time to nPSA. Only advanced T stage (HR 2.341; 95% CI 1.074–5.102; *P* = 0.032), nPSA with a cut-off of 0.06 ng/mL (HR 4.652; 95% CI 2.200–9.839; *P* < 0.001) and time to nPSA with a cut-off of 6 months (HR 2.211; 95% CI 1.019–4.796; *P* = 0.045) were significant while, GS (HR 1.629; 95% CI 0.715–3.711; *P* = 0.245), PSA at presentation (HR 1.053; 95% CI 0.494–2.243; *P* = 0.894) and age with a cut-off of 70 years (HR 1.039; 95% CI 0.485–2.228; *P* = 0.921) were not found to significantly affect bRFS. The lack of effect of baseline PSA and GS could be related to the fact that all patients were uniformly of the high-risk category. On multivariate analysis for bRFS, the only factor that maintained significance was nPSA (HR 3.804; 95% CI 1.689–8.567; *P* = 0.001).

For the subgroups (favorable and unfavorable), univariate analysis using bRFS as endpoint in the favorable group revealed two factors to have a significant impact: the use of ADT beyond 9 months, i.e. at least 12 months, (HR 0.224; 95% CI 0.070–0.720; *P* = 0.012) and time to achieve nPSA (tnPSA), dichotomized according to the median value of 6 months (HR 3.387; 95% CI 1.041–11.023; *P* = 0.043). The presenting T-stage was of borderline significance (HR 2.431; 95% CI 0.712–8.297; *P* = 0.056). Other factors such as PSA at presentation, dichotomized according to a cut-off value of 20 ng/mL, Gleason score dichotomized as ≤ 7 vs 8–10 and age, dichotomized according to a cut-off value of 70 years were non-significant. On multivariate analysis, factors that remained significant were the use of ADT beyond 9 months (HR 0.224; 95% CI 0.070–0.720; *P* = 0.012) and tnPSA (HR 4.369; 95% CI 1.096–17.421; *P* = 0.037); (Table 2). In the unfavorable group, univariate analysis for bRFS as endpoint revealed that the use of ADT beyond 12 months (i.e. at least 18 months) was the only significant factor (HR 0.303; 95% CI 0.105–0.876; *P* = 0.027); (Table 3). Multivariate analysis conducted for this group also showed the same findings (Table 3). It is important to note that since our groups are exclusively made of HR patients, they all had one, two, or all three of the HR factors (high T-stage, Gleason’s score > 7, and bPSA > 20). This could explain why those classical HR parameters were not found predictors of outcome, unlike what was found in our previous report where our patient population was composed of both high and intermediate-risk patients [16]. The finding that nPSA provides additional prognostic information in this select HR group patients is of significant importance as it provides an additional treatment-related prognostic factor.

**Table 2:** Predictors of biochemical recurrence-free survival in patients with Nadir PSA < 0.06 ng/mL

Factors	Univariate analysis		Multivariate analysis	
	HR (95% CI)	*P* value	HR (95% CI)	*P* value
Age (≤ 70 versus > 70 years)	0.530 (0.145–1.937)	0.337	–	–
T stage (T1-2 versus T3-4)	3.074 (0.972–9.719)	0.056	2.431 (0.712–8.297)	0.156
Gleason Score (≤ 7 versus 8–10)	1.045 (0.340–3.211)	0.939	–	–
PSA at presentation (≤ 20 ng/mL versus > 20 ng/mL)	1.088 (0.364–3.255)	0.880	–	–
Total ADT duration *	0.327 (0.109–0.978)	0.046	0.224 (0.070–0.720)	0.012
Time to nadir PSA (cut-off 6 months**)	3.387 (1.041–11.023)	0.043	4.369 (1.096–17.421)	0.037

*Comparing the use of ADT for at least 12 months versus 9 months or less

**Median time to nadir PSA

**Table 3:** Predictors of biochemical recurrence-free survival in patients with Nadir PSA ≥ 0.06 ng/mL

Factors	Univariate analysis		Multivariate analysis	
	HR (95% CI)	*P* value	HR (95% CI)	*P* value
Age (≤ 70 versus > 70 years)	1.248 (0.443–3.514)	0.675	1.178 (0.209–6.622)	0.853
T stage (T1-2 versus T3–4)	1.342 (0.461–3.908)	0.590	2.848 (0.515–15.747)	0.230
Gleason Score (≤ 7 versus 8–10)	2.118 (0.595–7.537)	0.247	3.018 (0.696–13.090)	0.140
PSA at presentation (≤ 20 versus > 20 ng/mL)	0.939 (0.310–2.840)	0.911	1.543 (0.412–5.787)	0.520
Total ADT duration*	0.319 (0.110–0.921)	0.035	0.306 (0.099–0.946)	0.040

*Comparing the use of ADT for at least 18 months versus less than 18 months

## Discussion

In a previous study we reported that nPSA levels correlate significantly with the biochemical recurrence outcomes of patients with IR or HR prostate cancer treated with combined definitive RT and ADT, with a nPSA cut-off of 0.06 ng/mL [16]. In the current analysis, we restricted the study group to those with HR disease, with the objective to examine the effect of duration of ADT for patients who achieved the desired nPSA and for those who did not. The duration of ADT varied between patients and depended on patient’s tolerance, compliance, as well as the treatment era in relation to existing standards, and/or physician’s preference based on disease burden. There was no correlation between the ADT duration and existing comorbidities. For patients who achieved the desired nPSA, the duration of ADT favorably affected biochemical outcome only when used up to 12 months. Beyond 12 months, there was no detectable benefit from prolonged ADT. For patients who did not reach nPSA of 0.06 ng/mL, the duration of ADT was found to have a favorable impact on biochemical recurrence up till 18 months; beyond 18 months, continuing ADT was not associated with a better outcome.

The optimal ADT duration in prostate cancer patients treated by definitive RT has been extensively studied over the past two decades [4–10, 19]. The RTOG 9408 study found that low risk patients did not benefit form combined ADT with definitive RT whereas IR patients fared better with definitive RT associated with a short course of 6 months of ADT [19]. The Radiation therapy and Oncology Group (RTOG) 9202 trial examining patients with T2c-T4 prostate cancer receiving 4 months of neoadjuvant and concurrent ADT with EBRT, randomized patients to no further ADT or an additional 2 years, and showed a significant improvement in all endpoints in the long-term ADT group [6]. Similarly, the European Organization for research and treatment of cancer (EORTC) 22961 trial showed that the combination of EBRT and 6 months of ADT provides inferior survival outcomes compared to a total of 3 years of ADT [4], and the DART01/05 GICOR trial showed that 24 months of ADT provided improved biochemical recurrence-free and overall survival compared with 4 months of ADT in patients with high risk localized prostate cancer [9].

However, delivering long term ADT is associated with several clinical and economical challenges. Patients who receive long term ADT are at increased risk for many complications such as anemia, osteoporosis, mood, mental, sexual, and musculoskeletal changes among others [11–14]. In addition, the cost of these drugs could be prohibitive, particularly in resource-limited settings. Identifying parameters that could define the subgroup of patients who would benefit the most from protracted ADT would optimize the use of these drugs and limit their side effects, particularly in older patients. Recent studies from Canada and Australia have examined the value of ADT for a total duration of 18 months and have shown that while 18 months of ADT was superior to 6 months of ADT [5], it was equivalent in survival outcomes to 36 months of ADT [7]. However, neither trial addressed whether some particular patients with favorable disease response could have a similarly favorable outcome with a lower duration of ADT. In our study, like in the Australian study, the use of ADT for 9 months or less was associated with lower bRFS. However, for those patients who achieved the desirable nPSA of 0.06 ng/mL, there was no added benefit to continue ADT beyond 12 months. On the other hand, for those who did not reach the desired nPSA, continuing ADT for a minimum of 18 months was superior to any shorter duration. Similar to the Canadian study, continuing ADT beyond 18 months for this subgroup was not associated with a better outcome.

The prognostic value of nPSA is well known but is not fully established [20–26]. A recent study from Memorial Sloan Kettering Cancer Center (MSKCC) reported similar findings to our results on a population of IR and HR patients treated by definitive RT and ADT and using PSA value at 3 months after EBRT. The authors found a PSA value of 0.09 ng/mL or lower to be highly predictive of outcome [22]. It is worth noting, that in our study and in several others, more classic prognostic factors like baseline PSA, radiation dose, T-stage, and sometimes Gleason score, have not been consistently found to be significant independent prognostic variables when examined in a multivariate regression model that includes nPSA [16, 24–28]. However, a recent meta-analysis evaluating the effect of ADT duration (short term, long term, or lifelong) by GS (8 vs. 9–10) has shown that patients with a higher GS are benefiting more from longer ADT duration [10]. In that study, GS was the only variable tested and this may explain the discrepancy with our results and that of others that included all parameters of HR in addition to nPSA. Our results and that of others using nPSA indicate that beyond tumor anatomic and biochemical characteristics, initial disease response is a strong prognostic indicator of disease outcome. This fact, if confirmed in prospective and other large studies, could impact on treatment individualization with a possibility of treatment de-escalation for those showing good treatment response and more escalation in those who do not [16, 22].

Another potential indicator of disease response in prostate cancer is time to nadir PSA (tnPSA). Previous studies have examined variable endpoints such as PSA level at given times after therapy and PSA decay kinetics [20, 22, 23, 26, 28–30]. In our study, tnPSA was found to have an impact on bRFS only in the favorable group that achieved the desired nPSA of 0.06 ng/mL. For those patients, if tnPSA is less that the median value of 6 months, the duration of ADT had no impact on outcomes suggesting that this favorable patient group may not need protracted ADT. It is important to note that these findings should be interpreted with caution given the low number of patients in this subgroup. In the study from MSKCC by Patel et al., PSA decay kinetics correlated well with outcome, not only for bRFS but also in distant metastasis-free survival (DMFS) [22]. Patients who had 95% or more decline in their PSA by 3 months after radiation had a better bRFS and a better DMFS than those who had less than 95% decline. Taken together, these data on nPSA and tnPSA after definitive RT and ADT could indicate a clinical possibility to de-escalate treatment for favorably responding patients and save those patients many of the undesirable side effects of ADT while maintaining a high level of disease control.

This is a retrospective analysis and as such, is subject to the inherent biases of such study designs. In addition, the study comprises a substantially smaller cohort of patients compared to the aforementioned randomized trails, limiting the generalizability of our findings. However, we believe that the findings are unique and could be hypothesis generating for larger prospective studies to gage the duration of ADT not only based on baseline risk categories but also on response to standard combined therapy, and examine the association between ADT duration, nPSA and cancer-specific survival. It is true that high risk factors such as the T stage, Gleason score and PSA at baseline were not found to correlate with a worse biochemical recurrence free survival. However, this could be related to the fact that our patient population contained only HR patients who had at least one and/or a combination of multiple HR parameters. Given that the cohort was restricted to high-risk patients, these findings were not surprising. Had intermediate risk prostate cancer patients been included in the analysis, a worse outcome would have been expected in patients with higher baseline PSA, higher Gleason score, and/or higher T stage.

## Conclusion

In conclusion, in this study on HR prostate cancer patients treated by definitive RT and ADT, parameters of PSA response like nPSA and tnPSA were found to be predictive of biochemical control and the extent of benefit from long term ADT. We observed that patients who achieved a nPSA of 0.06 ng/mL or less did not benefit from ADT beyond 12 months while those who did not achieve this nadir required at least 18 months of ADT. For those favorable patients who achieved the desired nadir, a short tnPSA ≤ 6 months may further indicate that short duration ADT could be sufficient. We believe that our results are hypothesis generating and could represent a stimulus for other groups to examine their databases and possibly initiate larger prospective studies to examine these parameters. If confirmed they could have the potential to change our risk classification and help clinicians adjust their adjunctive ADT based on these parameters of disease response.

## Data Availability

The datasets used and analyzed during the current study are available with author Zeina Ayoub, MD, who can be reached on za47@aub.edu.lb to obtain access to the data.

## Abbreviations

ADT: Androgen deprivation therapy
nPSA: Nadir prostate-specific antigen
bRFS: Biochemical recurrence-free survival
HR: High-risk
tnPSA: Time to nadir prostate-specific antigen
EBRT: External beam radiation therapy
LHRH: Luteinizing hormone-releasing hormone
IR: Intermediate-risk
IQR: Interquartile range
DMFS: Distant metastasis-free survival
